# *N* ^2^,*N* ^2^,*N* ^6^,*N* ^6^-Tetra­kis(2,3,4,5,6-penta­fluoro­benzo­yl)pyridine-2,6-diamine

**DOI:** 10.1107/S1600536811048768

**Published:** 2011-11-25

**Authors:** Arto Valkonen, Erkki Kolehmainen, Borys Ośmiałowski

**Affiliations:** a University of Jyväskylä, Department of Chemistry, PO Box 35, FIN-40014 Jyväskylä, Finland; b University of Technology and Life Sciences, Department of Chemistry, Seminaryjna 3, PL-85-326 Bydgoszcz, Poland

## Abstract

The title compound, C_33_H_3_F_20_N_3_O_4_, is a highly fluorinated organic imide that was isolated as an unexpected product from the reaction of 2,6-diamino­pyridine with 2,3,4,5,6-penta­fluoro­benzoyl chloride in a 1:2 molar ratio. The mol­ecule is located on a twofold axis and one of its symmetry-independent 2,3,4,5,6-penta­fluoro­benzoyl groups is disordered over two sets of sites, the occupancy of the major component being 0.773 (3). In the major component, the dihedral angle between the perfluoro­phenyl groups is 63.64 (10)°, and these groups form dihedral angles of 67.14 (7) and 21.1 (2)° with the pyridine core. Short inter­molecular C—H⋯O and C—H⋯N contacts are found in the crystal structure.

## Related literature

For preparation of 2-acyl­amino­pyridines and their structures, see: Ośmiałowski *et al.* (2010*a* ▶,*b* ▶). For related structures, see: Kovalevsky *et al.* (1999 ▶).
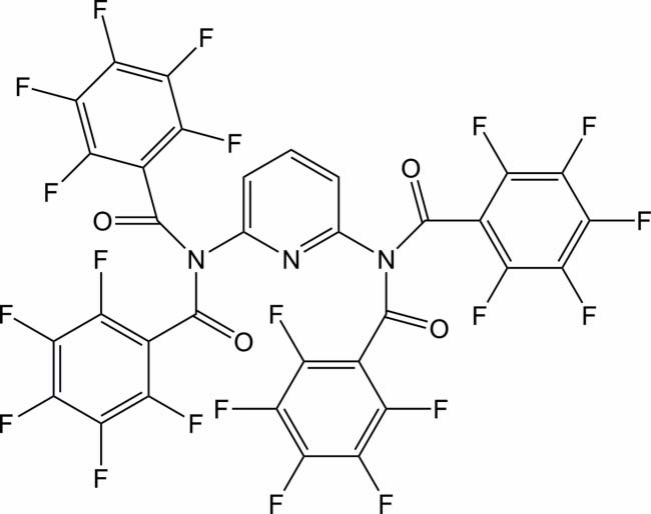


## Experimental

### 

#### Crystal data

C_33_H_3_F_20_N_3_O_4_
*M* *_r_* = 885.38
Monoclinic, 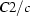

*a* = 21.2370 (5) Å
*b* = 6.3940 (1) Å
*c* = 23.1045 (5) Å
β = 100.585 (1)°
*V* = 3083.96 (11) Å^3^
*Z* = 4
Mo *K*α radiation
μ = 0.21 mm^−1^
*T* = 123 K
0.30 × 0.18 × 0.16 mm

#### Data collection

Bruker–Nonius KappaCCD with an APEXII detector diffractometer
7138 measured reflections
3785 independent reflections
2526 reflections with *I* > 2σ(*I*)
*R*_int_ = 0.039

#### Refinement

*R*[*F* ^2^ > 2σ(*F* ^2^)] = 0.057
*wR*(*F* ^2^) = 0.133
*S* = 1.05
3785 reflections
366 parameters
97 restraints
H-atom parameters constrained
Δρ_max_ = 0.35 e Å^−3^
Δρ_min_ = −0.42 e Å^−3^

### 

Data collection: *COLLECT* (Bruker, 2008 ▶); cell refinement: *DENZO-SMN* (Otwinowski & Minor, 1997 ▶); data reduction: *DENZO-SMN*; program(s) used to solve structure: *SIR2004* (Burla *et al.*, 2005 ▶); program(s) used to refine structure: *SHELXL97* (Sheldrick, 2008 ▶); molecular graphics: *ORTEP-3* (Farrugia, 1997 ▶) and *Mercury* (Macrae *et al.*, 2008 ▶); software used to prepare material for publication: *SHELXL97*.

## Supplementary Material

Crystal structure: contains datablock(s) global, I. DOI: 10.1107/S1600536811048768/gk2434sup1.cif

Structure factors: contains datablock(s) I. DOI: 10.1107/S1600536811048768/gk2434Isup2.hkl

Supplementary material file. DOI: 10.1107/S1600536811048768/gk2434Isup3.cml

Additional supplementary materials:  crystallographic information; 3D view; checkCIF report

## Figures and Tables

**Table 1:** Hydrogen-bond geometry (Å, °)

*D*—H⋯*A*	*D*—H	H⋯*A*	*D*⋯*A*	*D*—H⋯*A*
C4—H4⋯N1^i^	0.95	2.67	3.621 (5)	180
C3—H3⋯O1^i^	0.95	2.52	3.318 (3)	141

Symmetry code: (i) 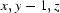.

**Fig. 1.**
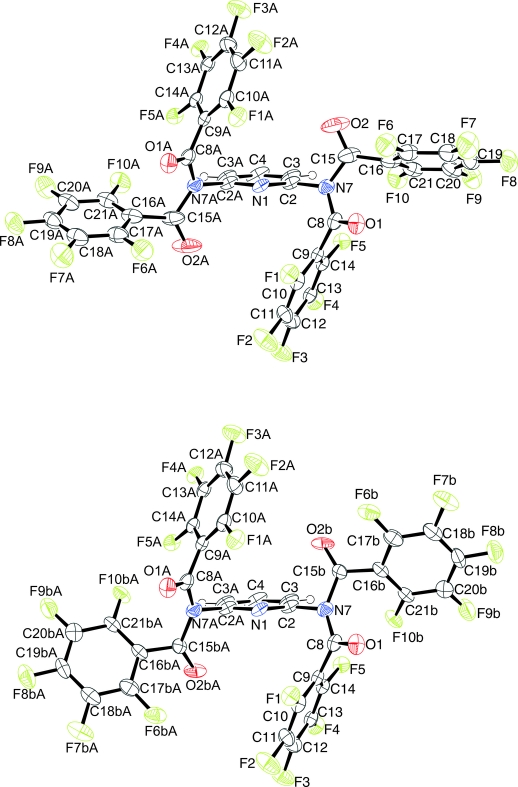
The structures of the major (top) and the minor (bottom) components of the disordered title molecule showing the atom-labeling scheme. Displacement ellipsoids are drawn at the 50% probability level. H atoms are represented by circles of arbitrary size.

**Fig. 2.**
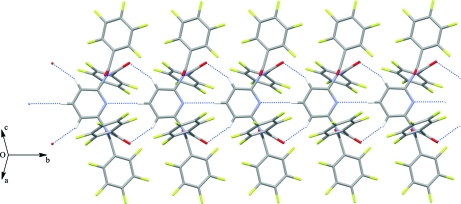
The short C—H···O/N contacts (blue dashed lines) organizing the molecules in the [0 1 0] direction. The minor component of disordered part of the molecule was removed for clarity.

## supplementary crystallographic information

### Comment

The title compound was accidentally obtained by reaction of 2,6-diaminopyridine with two equivalents of 2,3,4,5,6-pentafluorobenzoyl chloride, while the preparation of *N*,*N'*-bis(pentafluorobenzoyl)-2,6-diaminopyridine was attempted. The reaction was carried out analogously to our previously reported preparations of 2-acylaminopyridines (Ośmiałowski *et al.*, 2010*a*). Previously we have structurally characterized two related secondary amides (Ośmiałowski *et al.*, 2010*b*). This title imide has not been previously reported in the literature. The crystal structure of closely related imide, *N*,*N'*-(pyridine-2,6-diyl)-bis(naphthalenedicarboximide), has been reported (Kovalevsky *et al.*, 1999).

In the crystal molecules are located on a twofold rotation axis and one of the symmetry independent perfluorobenzoyl group is disordered over two sets of sites with different occupancies (Fig.1). The perfluorophenyl groups are twisted out of the pyridine core by 67.14 (7) and 21.1 (2) ° in the major component whereas in the minor component these angles are 67.14 (7) and 63.4 (4)°. Furthermore, the dihedral angle between perfluorophenyl - group planes is 63.64 (10) ° [the minor component 67.9 (4) °]. Mercury (Macrae *et al.*, 2008) helped us to find a motif along [0-10] direction (Fig. 2), where the molecule is connected to the translation related one by two C—H···O contacts (Table 1). Between these molecules there is also one rather long but linear C—H···N contact. A few F···C_Ar_, F···F and F···O type contacts were found, which are only slightly shorter than the sum of van der Waals radii.

### Experimental

2,3,4,5,6-Pentafluorobenzoyl chloride (2.28 g, 10 mmol) was added dropwise to a magnetically stirred solution of 2,6-diaminopyridine (0.54 g, 5 mmol) and triethylamine (1 ml) in dry methylene chloride (6 ml) at 0 °C. Subsequently the reaction mixture was refluxed for 4 h and the solution was treated with water and extracted with CH_2_Cl_2_. The organic solvent of the extract was evaporated under reduced pressure and the product recrystallized from hexane/ethyl acetate (10:1) mixture. ^1^H NMR (CDCl_3_): δ (p.p.m.) = 8.00 (t, 1H, H4), 7.45 (d, 2H, H3). Single crystals suitable for X-ray diffraction were obtained by very slow evaporation of analytical sample from NMR-tube, where CDCl_3_ was used as a solvent.

### Refinement

All H atoms were visible in electron density maps, but were calculated at their idealized positions and allowed to ride on their parent atoms at C—H distances of 0.95 Å with *U*_iso_(H) of 1.2 times *U*_eq_(C). A large number of restraints was needed to rationalize the disorder in perfluorobenzoyl group. FLAT (2 restraints, s=0.1) was applied to amide groups of both components to make them more planar. SADI (3 restraints, s=0.02) was applied for amide groups of both components to equalize the bond distances. DELU (2 restraints, s1=s2=0.01) was applied for one C—C and one C—F bond of the major component to equalize the anisotropic displacement parameters. SIMU (84 restraints, s=0.01, st=0.02, dmax=1.7) was applied to carbons of the disordered perfluorophenyl ring and carbonyl group of both components to equalize the anisotropic displacement parameters. ISOR (6 restraints, s=0.01, st=0.02) was applied to carbonyl oxygen atom of the minor component to prevent the atom to appear as non-positive definite.

### Crystal data

**Table:**

C_33_H_3_F_20_N_3_O_4_	*F*(000) = 1736
*M**_r_* = 885.38	*D*_x_ = 1.907 Mg m^−^^3^
Monoclinic, *C*2/*c*	Mo *K*α radiation, λ = 0.71073 Å
Hall symbol: -C 2yc	Cell parameters from 4030 reflections
*a* = 21.2370 (5) Å	θ = 0.4–28.3°
*b* = 6.3940 (1) Å	µ = 0.21 mm^−^^1^
*c* = 23.1045 (5) Å	*T* = 123 K
β = 100.585 (1)°	Block, colourless
*V* = 3083.96 (11) Å^3^	0.30 × 0.18 × 0.16 mm
*Z* = 4	

### Data collection

**Table:**

Bruker–Nonius KappaCCD with an APEXII detector diffractometer	2526 reflections with *I* > 2σ(*I*)
Radiation source: fine-focus sealed tube	*R*_int_ = 0.039
graphite	θ_max_ = 28.2°, θ_min_ = 1.8°
Detector resolution: 9 pixels mm^-1^	*h* = −27→28
φ and ω scans	*k* = −8→8
7138 measured reflections	*l* = −30→30
3785 independent reflections	

### Refinement

**Table:**

Refinement on *F*^2^	Primary atom site location: structure-invariant direct methods
Least-squares matrix: full	Secondary atom site location: difference Fourier map
*R*[*F*^2^ > 2σ(*F*^2^)] = 0.057	Hydrogen site location: inferred from neighbouring sites
*wR*(*F*^2^) = 0.133	H-atom parameters constrained
*S* = 1.05	*w* = 1/[σ^2^(*F*_o_^2^) + (0.0381*P*)^2^ + 7.2159*P*] where *P* = (*F*_o_^2^ + 2*F*_c_^2^)/3
3785 reflections	(Δ/σ)_max_ < 0.001
366 parameters	Δρ_max_ = 0.35 e Å^−^^3^
97 restraints	Δρ_min_ = −0.42 e Å^−^^3^

### Special details

**Table:**

Experimental. ^13^C NMR (CDCl_3_): δ (ppm) = 159.4, 149.1, 144.5, 142.4, 141.7, 138.7, 136.6, 123.8, 110.5.
Geometry. All s.u.'s (except the s.u. in the dihedral angle between two l.s. planes) are estimated using the full covariance matrix. The cell s.u.'s are taken into account individually in the estimation of s.u.'s in distances, angles and torsion angles; correlations between s.u.'s in cell parameters are only used when they are defined by crystal symmetry. An approximate (isotropic) treatment of cell s.u.'s is used for estimating s.u.'s involving l.s. planes.
Refinement. Refinement of *F*^2^ against ALL reflections. The weighted *R*-factor *wR* and goodness of fit *S* are based on *F*^2^, conventional *R*-factors *R* are based on *F*, with *F* set to zero for negative *F*^2^. The threshold expression of *F*^2^ > 2σ(*F*^2^) is used only for calculating *R*-factors(gt) *etc*. and is not relevant to the choice of reflections for refinement. *R*-factors based on *F*^2^ are statistically about twice as large as those based on *F*, and *R*- factors based on ALL data will be even larger.

### Fractional atomic coordinates and isotropic or equivalent isotropic displacement parameters (Å^2^)

**Table:**

	*x*	*y*	*z*	*U*_iso_*/*U*_eq_	Occ. (<1)
F1	0.86727 (7)	0.9378 (2)	0.69841 (7)	0.0376 (4)	
F2	0.78577 (10)	0.8370 (3)	0.59909 (7)	0.0616 (5)	
F3	0.72661 (9)	0.4583 (3)	0.58930 (7)	0.0579 (5)	
F4	0.75118 (6)	0.1782 (2)	0.67885 (7)	0.0362 (4)	
F5	0.82680 (7)	0.2875 (2)	0.78079 (6)	0.0345 (4)	
O1	0.87489 (9)	0.8475 (3)	0.82339 (8)	0.0380 (4)	
N7	0.93905 (9)	0.5589 (3)	0.82488 (9)	0.0330 (5)	
N1	1.0000	0.5463 (4)	0.7500	0.0278 (6)	
C2	0.97130 (10)	0.4368 (4)	0.78664 (11)	0.0303 (5)	
C3	0.97017 (12)	0.2207 (4)	0.78901 (14)	0.0392 (7)	
H3	0.9496	0.1495	0.8165	0.047*	
C4	1.0000	0.1126 (6)	0.7500	0.0450 (11)	
H4	1.0000	−0.0360	0.7500	0.054*	
C8	0.88798 (11)	0.6875 (4)	0.80002 (11)	0.0295 (5)	
C9	0.84960 (10)	0.6158 (4)	0.74248 (10)	0.0253 (5)	
C10	0.83779 (11)	0.7515 (4)	0.69504 (11)	0.0293 (5)	
C11	0.79688 (13)	0.6999 (4)	0.64361 (11)	0.0373 (6)	
C12	0.76727 (13)	0.5075 (4)	0.63878 (11)	0.0368 (6)	
C13	0.77919 (11)	0.3668 (4)	0.68450 (11)	0.0294 (5)	
C14	0.81941 (10)	0.4222 (4)	0.73576 (10)	0.0257 (5)	
O2	1.02137 (19)	0.5421 (8)	0.9007 (2)	0.0851 (16)	0.773 (2)
C15	0.9663 (3)	0.5901 (8)	0.8850 (2)	0.0484 (16)	0.773 (2)
F6	0.96821 (11)	0.9929 (4)	0.93716 (10)	0.0545 (6)	0.773 (2)
F7	0.90327 (13)	1.0815 (4)	1.02541 (11)	0.0641 (8)	0.773 (2)
F8	0.82644 (14)	0.7899 (4)	1.06022 (10)	0.0548 (7)	0.773 (2)
F9	0.81043 (14)	0.4137 (4)	1.00624 (12)	0.0565 (7)	0.773 (2)
F10	0.87435 (13)	0.3237 (4)	0.91753 (12)	0.0533 (7)	0.773 (2)
C16	0.92394 (13)	0.6546 (4)	0.92708 (10)	0.0413 (10)	0.773 (2)
C17	0.93101 (11)	0.8490 (4)	0.95442 (11)	0.0429 (9)	0.773 (2)
C18	0.89756 (12)	0.8949 (3)	0.99922 (10)	0.0451 (9)	0.773 (2)
C19	0.85705 (12)	0.7464 (4)	1.01668 (10)	0.0432 (9)	0.773 (2)
C20	0.84999 (14)	0.5520 (4)	0.98934 (13)	0.0407 (11)	0.773 (2)
C21	0.88343 (15)	0.5061 (3)	0.94454 (13)	0.0381 (12)	0.773 (2)
O2B	1.0322 (5)	0.4514 (16)	0.8935 (5)	0.041 (3)	0.227 (2)
C15B	0.9785 (6)	0.524 (2)	0.8821 (5)	0.027 (4)	0.227 (2)
F6B	1.0454 (3)	0.7631 (12)	0.9763 (3)	0.048 (2)	0.227 (2)
F7B	0.9953 (4)	0.8597 (13)	1.0711 (3)	0.060 (2)	0.227 (2)
F8B	0.8734 (5)	0.7549 (14)	1.0761 (3)	0.059 (2)	0.227 (2)
F9B	0.8023 (5)	0.5329 (19)	0.9872 (4)	0.058 (3)	0.227 (2)
F10B	0.8522 (4)	0.4215 (13)	0.8935 (3)	0.0397 (18)	0.227 (2)
C16B	0.9465 (4)	0.6015 (14)	0.9305 (3)	0.029 (3)	0.227 (2)
C17B	0.9844 (3)	0.7046 (14)	0.9773 (3)	0.040 (2)	0.227 (2)
C18B	0.9591 (4)	0.7582 (14)	1.0267 (3)	0.043 (3)	0.227 (2)
C19B	0.8959 (4)	0.7086 (15)	1.0293 (3)	0.042 (3)	0.227 (2)
C20B	0.8581 (3)	0.6055 (17)	0.9825 (4)	0.034 (3)	0.227 (2)
C21B	0.8833 (4)	0.5519 (16)	0.9331 (4)	0.025 (3)	0.227 (2)

### Atomic displacement parameters (Å^2^)

**Table:**

	*U*^11^	*U*^22^	*U*^33^	*U*^12^	*U*^13^	*U*^23^
F1	0.0398 (8)	0.0247 (7)	0.0498 (9)	−0.0086 (6)	0.0121 (7)	0.0033 (7)
F2	0.0912 (14)	0.0489 (11)	0.0402 (9)	−0.0188 (10)	0.0006 (9)	0.0136 (8)
F3	0.0692 (12)	0.0568 (12)	0.0403 (9)	−0.0210 (10)	−0.0093 (8)	−0.0015 (9)
F4	0.0296 (7)	0.0285 (8)	0.0512 (9)	−0.0101 (6)	0.0095 (6)	−0.0078 (7)
F5	0.0346 (7)	0.0277 (8)	0.0409 (8)	−0.0072 (6)	0.0062 (6)	0.0061 (6)
O1	0.0464 (11)	0.0271 (9)	0.0434 (10)	−0.0083 (8)	0.0159 (8)	−0.0102 (8)
N7	0.0269 (10)	0.0343 (12)	0.0373 (11)	−0.0054 (9)	0.0049 (9)	−0.0024 (10)
N1	0.0174 (12)	0.0214 (14)	0.0433 (17)	0.000	0.0021 (12)	0.000
C2	0.0179 (10)	0.0263 (12)	0.0447 (14)	−0.0012 (10)	0.0000 (10)	0.0012 (11)
C3	0.0236 (12)	0.0271 (13)	0.0656 (19)	−0.0033 (10)	0.0042 (12)	0.0084 (13)
C4	0.0240 (17)	0.0192 (17)	0.089 (3)	0.000	0.0020 (19)	0.000
C8	0.0276 (11)	0.0245 (12)	0.0386 (13)	−0.0081 (10)	0.0118 (10)	−0.0014 (11)
C9	0.0219 (10)	0.0237 (11)	0.0331 (12)	−0.0003 (9)	0.0118 (9)	−0.0031 (10)
C10	0.0304 (12)	0.0201 (11)	0.0400 (14)	−0.0056 (10)	0.0140 (11)	−0.0031 (11)
C11	0.0457 (15)	0.0356 (15)	0.0319 (13)	−0.0049 (12)	0.0105 (11)	0.0059 (12)
C12	0.0383 (14)	0.0381 (15)	0.0329 (13)	−0.0081 (12)	0.0033 (11)	−0.0051 (12)
C13	0.0256 (11)	0.0241 (12)	0.0408 (14)	−0.0059 (10)	0.0121 (10)	−0.0057 (11)
C14	0.0228 (10)	0.0230 (12)	0.0330 (12)	0.0016 (9)	0.0095 (9)	−0.0003 (10)
O2	0.040 (2)	0.106 (4)	0.094 (3)	0.032 (2)	−0.0279 (18)	−0.061 (3)
C15	0.035 (2)	0.041 (3)	0.062 (3)	0.009 (2)	−0.011 (2)	−0.026 (2)
F6	0.0586 (14)	0.0444 (14)	0.0607 (15)	−0.0048 (12)	0.0116 (12)	−0.0191 (12)
F7	0.0776 (17)	0.0436 (14)	0.0734 (17)	−0.0001 (13)	0.0204 (13)	−0.0319 (13)
F8	0.0691 (17)	0.0515 (15)	0.0451 (13)	0.0136 (13)	0.0142 (12)	−0.0149 (11)
F9	0.0761 (18)	0.0438 (16)	0.0508 (16)	0.0007 (15)	0.0151 (14)	−0.0041 (13)
F10	0.0680 (17)	0.0360 (14)	0.0561 (16)	0.0013 (12)	0.0117 (13)	−0.0186 (12)
C16	0.038 (2)	0.036 (2)	0.043 (2)	0.0126 (18)	−0.0102 (16)	−0.0140 (17)
C17	0.0409 (18)	0.0379 (19)	0.045 (2)	0.0075 (16)	−0.0048 (16)	−0.0116 (17)
C18	0.050 (2)	0.0365 (19)	0.044 (2)	0.0138 (17)	−0.0044 (16)	−0.0166 (17)
C19	0.047 (2)	0.044 (2)	0.0341 (18)	0.0151 (18)	−0.0019 (16)	−0.0082 (17)
C20	0.055 (3)	0.029 (2)	0.033 (2)	0.0114 (19)	−0.006 (2)	−0.0009 (18)
C21	0.048 (2)	0.023 (2)	0.037 (2)	0.0125 (17)	−0.0080 (18)	−0.0023 (18)
O2B	0.040 (5)	0.030 (5)	0.044 (5)	0.004 (4)	−0.016 (4)	0.017 (4)
C15B	0.030 (8)	0.027 (8)	0.024 (6)	−0.005 (6)	0.005 (5)	0.009 (6)
F6B	0.048 (4)	0.050 (5)	0.040 (4)	−0.003 (4)	−0.007 (3)	0.011 (4)
F7B	0.083 (6)	0.049 (5)	0.038 (4)	0.001 (5)	−0.012 (4)	−0.003 (4)
F8B	0.077 (6)	0.065 (6)	0.038 (4)	0.022 (5)	0.020 (4)	−0.007 (4)
F9B	0.067 (6)	0.076 (7)	0.036 (5)	−0.012 (6)	0.018 (4)	−0.005 (5)
F10B	0.055 (5)	0.038 (4)	0.025 (3)	−0.013 (4)	0.004 (3)	−0.006 (3)
C16B	0.042 (6)	0.023 (5)	0.020 (4)	0.001 (5)	0.002 (4)	0.000 (4)
C17B	0.053 (6)	0.031 (5)	0.032 (5)	−0.001 (5)	0.000 (4)	0.003 (4)
C18B	0.060 (6)	0.039 (5)	0.027 (5)	0.007 (5)	−0.001 (5)	−0.002 (4)
C19B	0.059 (6)	0.045 (6)	0.024 (5)	0.006 (5)	0.015 (5)	−0.001 (4)
C20B	0.046 (6)	0.021 (5)	0.037 (5)	0.005 (5)	0.009 (5)	0.014 (5)
C21B	0.044 (6)	0.009 (4)	0.020 (5)	0.003 (4)	0.001 (4)	0.002 (4)

### Geometric parameters (Å, °)

**Table:**

F1—C10	1.341 (3)	C15—C16	1.498 (6)
F2—C11	1.339 (3)	F6—C17	1.321 (3)
F3—C12	1.338 (3)	F7—C18	1.333 (3)
F4—C13	1.340 (3)	F8—C19	1.324 (3)
F5—C14	1.338 (3)	F9—C20	1.327 (4)
O1—C8	1.213 (3)	F10—C21	1.320 (3)
N7—C8	1.398 (3)	C16—C17	1.3900
N7—C15	1.417 (6)	C16—C21	1.3900
N7—C2	1.443 (3)	C17—C18	1.3900
N7—C15B	1.446 (12)	C18—C19	1.3900
N1—C2^i^	1.330 (3)	C19—C20	1.3900
N1—C2	1.330 (3)	C20—C21	1.3900
C2—C3	1.383 (4)	O2B—C15B	1.215 (14)
C3—C4	1.379 (3)	C15B—C16B	1.497 (13)
C3—H3	0.9500	F6B—C17B	1.353 (9)
C4—C3^i^	1.379 (3)	F7B—C18B	1.332 (10)
C4—H4	0.9500	F8B—C19B	1.294 (9)
C8—C9	1.498 (3)	F9B—C20B	1.294 (12)
C9—C10	1.384 (3)	F10B—C21B	1.321 (9)
C9—C14	1.389 (3)	C16B—C17B	1.3900
C10—C11	1.377 (4)	C16B—C21B	1.3900
C11—C12	1.377 (4)	C17B—C18B	1.3900
C12—C13	1.375 (4)	C18B—C19B	1.3900
C13—C14	1.374 (3)	C19B—C20B	1.3900
O2—C15	1.200 (6)	C20B—C21B	1.3900
			
C8—N7—C15	117.8 (3)	C17—C16—C21	120.0
C8—N7—C2	119.1 (2)	C17—C16—C15	120.9 (3)
C15—N7—C2	121.5 (3)	C21—C16—C15	118.5 (3)
C8—N7—C15B	138.0 (6)	F6—C17—C16	120.4 (2)
C2—N7—C15B	102.4 (6)	F6—C17—C18	119.6 (2)
C2^i^—N1—C2	116.5 (3)	C16—C17—C18	120.0
N1—C2—C3	124.4 (3)	F7—C18—C19	119.3 (2)
N1—C2—N7	115.5 (2)	F7—C18—C17	120.7 (2)
C3—C2—N7	120.2 (2)	C19—C18—C17	120.0
C4—C3—C2	117.5 (3)	F8—C19—C18	119.6 (2)
C4—C3—H3	121.3	F8—C19—C20	120.4 (2)
C2—C3—H3	121.3	C18—C19—C20	120.0
C3—C4—C3^i^	119.8 (4)	F9—C20—C21	121.1 (2)
C3—C4—H4	120.1	F9—C20—C19	118.9 (2)
C3^i^—C4—H4	120.1	C21—C20—C19	120.0
O1—C8—N7	122.5 (2)	F10—C21—C20	119.2 (2)
O1—C8—C9	121.4 (2)	F10—C21—C16	120.7 (2)
N7—C8—C9	116.1 (2)	C20—C21—C16	120.0
C10—C9—C14	117.2 (2)	O2B—C15B—N7	128.4 (11)
C10—C9—C8	120.1 (2)	O2B—C15B—C16B	120.1 (11)
C14—C9—C8	122.4 (2)	N7—C15B—C16B	111.4 (9)
F1—C10—C11	118.5 (2)	C17B—C16B—C21B	120.0
F1—C10—C9	119.7 (2)	C17B—C16B—C15B	117.5 (7)
C11—C10—C9	121.8 (2)	C21B—C16B—C15B	122.0 (7)
F2—C11—C12	120.6 (2)	F6B—C17B—C18B	117.5 (6)
F2—C11—C10	120.0 (2)	F6B—C17B—C16B	122.5 (6)
C12—C11—C10	119.4 (2)	C18B—C17B—C16B	120.0
F3—C12—C13	120.1 (2)	F7B—C18B—C17B	119.8 (7)
F3—C12—C11	119.5 (2)	F7B—C18B—C19B	120.2 (7)
C13—C12—C11	120.4 (2)	C17B—C18B—C19B	120.0
F4—C13—C14	120.7 (2)	F8B—C19B—C20B	120.6 (8)
F4—C13—C12	119.9 (2)	F8B—C19B—C18B	119.4 (8)
C14—C13—C12	119.4 (2)	C20B—C19B—C18B	120.0
F5—C14—C13	118.0 (2)	F9B—C20B—C19B	120.8 (8)
F5—C14—C9	120.1 (2)	F9B—C20B—C21B	118.5 (8)
C13—C14—C9	121.8 (2)	C19B—C20B—C21B	120.0
O2—C15—N7	117.4 (5)	F10B—C21B—C20B	120.3 (7)
O2—C15—C16	122.8 (5)	F10B—C21B—C16B	118.6 (7)
N7—C15—C16	119.3 (4)	C20B—C21B—C16B	120.0
			
C2^i^—N1—C2—C3	0.57 (19)	C21—C16—C17—C18	0.0
C2^i^—N1—C2—N7	−178.4 (2)	C15—C16—C17—C18	−171.1 (3)
C8—N7—C2—N1	62.2 (3)	F6—C17—C18—F7	−0.9 (3)
C15—N7—C2—N1	−103.0 (4)	C16—C17—C18—F7	−179.2 (3)
C15B—N7—C2—N1	−111.0 (6)	F6—C17—C18—C19	178.4 (3)
C8—N7—C2—C3	−116.9 (3)	C16—C17—C18—C19	0.0
C15—N7—C2—C3	78.0 (4)	F7—C18—C19—F8	−2.2 (3)
C15B—N7—C2—C3	70.0 (6)	C17—C18—C19—F8	178.6 (3)
N1—C2—C3—C4	−1.1 (4)	F7—C18—C19—C20	179.3 (3)
N7—C2—C3—C4	177.85 (18)	C17—C18—C19—C20	0.0
C2—C3—C4—C3^i^	0.50 (17)	F8—C19—C20—F9	2.3 (3)
C15—N7—C8—O1	15.2 (4)	C18—C19—C20—F9	−179.1 (3)
C2—N7—C8—O1	−150.5 (2)	F8—C19—C20—C21	−178.6 (3)
C15B—N7—C8—O1	19.5 (9)	C18—C19—C20—C21	0.0
C15—N7—C8—C9	−164.5 (3)	F9—C20—C21—F10	1.7 (3)
C2—N7—C8—C9	29.8 (3)	C19—C20—C21—F10	−177.5 (3)
C15B—N7—C8—C9	−160.2 (8)	F9—C20—C21—C16	179.1 (3)
O1—C8—C9—C10	51.4 (3)	C19—C20—C21—C16	0.0
N7—C8—C9—C10	−128.9 (2)	C17—C16—C21—F10	177.4 (3)
O1—C8—C9—C14	−122.1 (3)	C15—C16—C21—F10	−11.2 (4)
N7—C8—C9—C14	57.6 (3)	C17—C16—C21—C20	0.0
C14—C9—C10—F1	−177.86 (19)	C15—C16—C21—C20	171.3 (3)
C8—C9—C10—F1	8.3 (3)	C8—N7—C15B—O2B	−155.9 (9)
C14—C9—C10—C11	1.3 (4)	C2—N7—C15B—O2B	15.1 (14)
C8—C9—C10—C11	−172.6 (2)	C8—N7—C15B—C16B	20.5 (14)
F1—C10—C11—F2	−2.1 (4)	C2—N7—C15B—C16B	−168.5 (7)
C9—C10—C11—F2	178.8 (2)	O2B—C15B—C16B—C17B	37.7 (14)
F1—C10—C11—C12	178.5 (2)	N7—C15B—C16B—C17B	−139.0 (8)
C9—C10—C11—C12	−0.6 (4)	O2B—C15B—C16B—C21B	−134.5 (10)
F2—C11—C12—F3	−0.6 (4)	N7—C15B—C16B—C21B	48.8 (11)
C10—C11—C12—F3	178.8 (3)	C21B—C16B—C17B—F6B	−177.6 (9)
F2—C11—C12—C13	179.6 (2)	C15B—C16B—C17B—F6B	10.0 (10)
C10—C11—C12—C13	−1.1 (4)	C21B—C16B—C17B—C18B	0.0
F3—C12—C13—F4	1.6 (4)	C15B—C16B—C17B—C18B	−172.3 (9)
C11—C12—C13—F4	−178.6 (2)	F6B—C17B—C18B—F7B	−1.2 (9)
F3—C12—C13—C14	−177.9 (2)	C16B—C17B—C18B—F7B	−179.0 (9)
C11—C12—C13—C14	2.0 (4)	F6B—C17B—C18B—C19B	177.8 (9)
F4—C13—C14—F5	−3.7 (3)	C16B—C17B—C18B—C19B	0.0
C12—C13—C14—F5	175.7 (2)	F7B—C18B—C19B—F8B	−2.6 (11)
F4—C13—C14—C9	179.26 (19)	C17B—C18B—C19B—F8B	178.4 (10)
C12—C13—C14—C9	−1.3 (4)	F7B—C18B—C19B—C20B	179.0 (9)
C10—C9—C14—F5	−177.3 (2)	C17B—C18B—C19B—C20B	0.0
C8—C9—C14—F5	−3.6 (3)	F8B—C19B—C20B—F9B	−7.9 (12)
C10—C9—C14—C13	−0.3 (3)	C18B—C19B—C20B—F9B	170.5 (12)
C8—C9—C14—C13	173.4 (2)	F8B—C19B—C20B—C21B	−178.4 (10)
C8—N7—C15—O2	−153.1 (5)	C18B—C19B—C20B—C21B	0.0
C2—N7—C15—O2	12.2 (7)	F9B—C20B—C21B—F10B	−3.3 (10)
C8—N7—C15—C16	34.5 (6)	C19B—C20B—C21B—F10B	167.5 (10)
C2—N7—C15—C16	−160.2 (3)	F9B—C20B—C21B—C16B	−170.7 (11)
O2—C15—C16—C17	73.5 (6)	C19B—C20B—C21B—C16B	0.0
N7—C15—C16—C17	−114.5 (4)	C17B—C16B—C21B—F10B	−167.7 (10)
O2—C15—C16—C21	−97.7 (6)	C15B—C16B—C21B—F10B	4.3 (11)
N7—C15—C16—C21	74.2 (5)	C17B—C16B—C21B—C20B	0.0
C21—C16—C17—F6	−178.4 (3)	C15B—C16B—C21B—C20B	172.0 (9)
C15—C16—C17—F6	10.5 (4)		

Symmetry codes: (i) −*x*+2, *y*, −*z*+3/2.

### Hydrogen-bond geometry (Å, °)

**Table:**

*D*—H···*A*	*D*—H	H···*A*	*D*···*A*	*D*—H···*A*
C4—H4···N1^ii^	0.95	2.67	3.621 (5)	180
C3—H3···O1^ii^	0.95	2.52	3.318 (3)	141

Symmetry codes: (ii) *x*, *y*−1, *z*.
